# [(2-Morpholinoeth­yl)(2-pyridylmethyl­ene)amine]dithio­cyanato­zinc(II)

**DOI:** 10.1107/S1600536808044061

**Published:** 2009-01-08

**Authors:** Bang-Hong Cai

**Affiliations:** a Department of Chemistry, Jiaying University, Meizhou Guangdong 514015, People’s Republic of China

## Abstract

The title compound, [Zn(NCS)_2_(C_12_H_17_N_3_O)], was prepared by the reaction of zinc acetate with pyridine-2-carbaldehyde, 2-morpholinoethyl­amine and ammonium thio­cyanate in an ethanol solution. The Zn^II^ atom is five coordinate with a distorted trigonal–bipyramidal geometry, coordinating with three N atoms of the Schiff base (2-morpholinoeth­yl)(2-pyridylmethyl­idene)amine and two N atoms from two thio­cyanate ligands. The morpholine ring adopts a chair configuration.

## Related literature

For background literature on Schiff base complexes, see: Costes *et al.* (2002 ▶); Erxleben (2001 ▶); Lacroix *et al.* (1996 ▶); Odoko *et al.* (2006 ▶); Ali *et al.* (2006 ▶). For literature on related zinc(II) complexes, see: Li *et al.* (2008 ▶); Eltayeb *et al.* (2007 ▶); Ali *et al.* (2008 ▶); Zhang & Wang (2007 ▶).
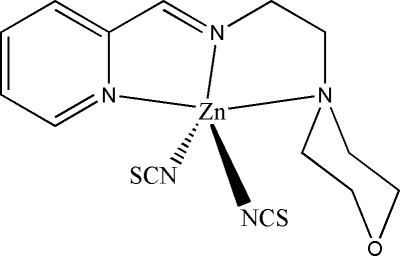


## Experimental

### 

#### Crystal data

[Zn(NCS)_2_(C_12_H_17_N_3_O)]
*M* *_r_* = 400.82
Triclinic, 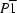

*a* = 8.185 (2) Å
*b* = 8.654 (2) Å
*c* = 13.368 (4) Å
α = 98.439 (3)°
β = 102.587 (3)°
γ = 102.501 (3)°
*V* = 883.3 (4) Å^3^
*Z* = 2
Mo *K*α radiation
μ = 1.64 mm^−1^
*T* = 298 (2) K
0.23 × 0.23 × 0.20 mm

#### Data collection

Bruker SMART 1000 CCD area-detector diffractometer
Absorption correction: multi-scan (*SADABS*; Sheldrick, 1996 ▶) *T*_min_ = 0.705, *T*_max_ = 0.736
7386 measured reflections
3770 independent reflections
2989 reflections with *I* > 2σ(*I*)
*R*_int_ = 0.031

#### Refinement

*R*[*F* ^2^ > 2σ(*F* ^2^)] = 0.045
*wR*(*F* ^2^) = 0.124
*S* = 1.04
3770 reflections
208 parameters
H-atom parameters constrained
Δρ_max_ = 0.57 e Å^−3^
Δρ_min_ = −0.47 e Å^−3^

### 

Data collection: *SMART* (Bruker, 2002 ▶); cell refinement: *SAINT* (Bruker, 2002 ▶); data reduction: *SAINT*; program(s) used to solve structure: *SHELXS97* (Sheldrick, 2008 ▶); program(s) used to refine structure: *SHELXL97* (Sheldrick, 2008 ▶); molecular graphics: *SHELXTL* (Sheldrick, 2008 ▶); software used to prepare material for publication: *SHELXTL*.

## Supplementary Material

Crystal structure: contains datablocks global, I. DOI: 10.1107/S1600536808044061/su2087sup1.cif

Structure factors: contains datablocks I. DOI: 10.1107/S1600536808044061/su2087Isup2.hkl

Additional supplementary materials:  crystallographic information; 3D view; checkCIF report

## Figures and Tables

**Fig. 1.**
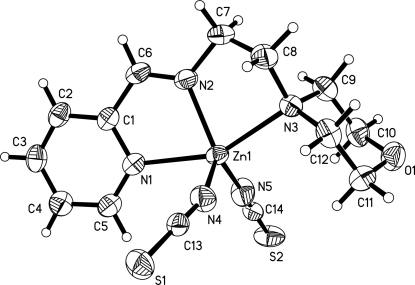
The molecular structure of the compound (I), showing 30% probability displacement ellipsoids.

## supplementary crystallographic information

### Comment

Schiff bases are extremely interesting ligands and many have been used to form a large number of metal complexes (Costes *et al.*, 2002; Erxleben, 2001; Lacroix *et al.*, 1996; Odoko *et al.*, 2006; Ali *et al.*, 2006). As a continuation of our work in this area, we report herein the crystal structure of a new zinc(II) complex of the Schiff base (2-morpholin-4-ylethyl)-(1-pyridin-2-ylmethylidene)amine and ammonium thiocyanate, (I).

The molecular structure of complex (I) is illustrated in Fig. 1. The Zn^II^ atom is five-coordinate in a trigonal-bipyramidal geometry, coordinating with three N-atoms of the Schiff base ligand and two N-atoms from two thiocyanate ligands. All the coordinate bond lengths are typical and comparable with those in the similar zinc(II) complexes (Li *et al.*, 2008; Eltayeb *et al.*, 2007; Ali *et al.*, 2008; Zhang & Wang, 2007). As expected, the morpholine ring adopts a chair configuration.

### Experimental

Pyridine-2-carbaldehyde (0.1 mmol, 10.7 mg), 2-morpholin-4-ylethylamine (0.1 mmol, 13.0 mg), ammonium thiocyanate (0.2 mmol, 15.2 mg), and zinc acetate dihydrate (0.1 mmol, 22.0 mg) were mixed in an ethanol solution (20 ml). The mixture was stirred for 2 h at room temperature, giving a colorless solution. Single-crystals were formed by gradual evaporation of the solution in air after several days.

### Refinement

H atoms were placed in calculated positions and treated as riding atoms: C–H = 0.93 - 0.97 Å, with *U*_iso_(H) = 1.2*U*_eq_(C).

### Crystal data

**Table:**

[Zn(NCS)_2_(C_12_H_17_N_3_O)]	*Z* = 2
*M**_r_* = 400.82	*F*(000) = 412
Triclinic, *P*1	*D*_x_ = 1.507 Mg m^−^^3^
Hall symbol: -P 1	Mo *K*α radiation, λ = 0.71073 Å
*a* = 8.185 (2) Å	Cell parameters from 2675 reflections
*b* = 8.654 (2) Å	θ = 2.4–25.0°
*c* = 13.368 (4) Å	µ = 1.64 mm^−^^1^
α = 98.439 (3)°	*T* = 298 K
β = 102.587 (3)°	Block, colorless
γ = 102.501 (3)°	0.23 × 0.23 × 0.20 mm
*V* = 883.3 (4) Å^3^	

### Data collection

**Table:**

Bruker SMART 1000 CCD area-detector diffractometer	3770 independent reflections
Radiation source: fine-focus sealed tube	2989 reflections with *I* > 2σ(*I*)
graphite	*R*_int_ = 0.031
ω scans	θ_max_ = 27.0°, θ_min_ = 1.6°
Absorption correction: multi-scan (*SADABS*; Sheldrick, 1996)	*h* = −10→10
*T*_min_ = 0.705, *T*_max_ = 0.736	*k* = −11→10
7386 measured reflections	*l* = −17→16

### Refinement

**Table:**

Refinement on *F*^2^	Primary atom site location: structure-invariant direct methods
Least-squares matrix: full	Secondary atom site location: difference Fourier map
*R*[*F*^2^ > 2σ(*F*^2^)] = 0.045	Hydrogen site location: inferred from neighbouring sites
*wR*(*F*^2^) = 0.124	H-atom parameters constrained
*S* = 1.04	*w* = 1/[σ^2^(*F*_o_^2^) + (0.064*P*)^2^ + 0.1459*P*] where *P* = (*F*_o_^2^ + 2*F*_c_^2^)/3
3770 reflections	(Δ/σ)_max_ = 0.001
208 parameters	Δρ_max_ = 0.57 e Å^−^^3^
0 restraints	Δρ_min_ = −0.47 e Å^−^^3^

### Special details

**Table:**

Geometry. All esds (except the esd in the dihedral angle between two l.s. planes) are estimated using the full covariance matrix. The cell esds are taken into account individually in the estimation of esds in distances, angles and torsion angles; correlations between esds in cell parameters are only used when they are defined by crystal symmetry. An approximate (isotropic) treatment of cell esds is used for estimating esds involving l.s. planes.
Refinement. Refinement of F^2^ against ALL reflections. The weighted R-factor wR and goodness of fit S are based on F^2^, conventional R-factors R are based on F, with F set to zero for negative F^2^. The threshold expression of F^2^ > 2sigma(F^2^) is used only for calculating R-factors(gt) etc. and is not relevant to the choice of reflections for refinement. R-factors based on F^2^ are statistically about twice as large as those based on F, and R- factors based on ALL data will be even larger.

### Fractional atomic coordinates and isotropic or equivalent isotropic displacement parameters (Å^2^)

**Table:**

	*x*	*y*	*z*	*U*_iso_*/*U*_eq_	
Zn1	0.87080 (4)	0.35121 (4)	0.23426 (3)	0.04844 (15)	
S1	0.75132 (16)	0.05952 (14)	−0.10471 (7)	0.0759 (3)	
S2	1.40376 (14)	0.73452 (15)	0.38080 (9)	0.0854 (4)	
O1	1.1836 (4)	0.1269 (4)	0.4455 (2)	0.0791 (8)	
N1	0.7747 (4)	0.5343 (3)	0.1472 (2)	0.0551 (7)	
N2	0.6437 (3)	0.3649 (3)	0.2720 (2)	0.0552 (7)	
N3	0.8546 (3)	0.1714 (3)	0.3427 (2)	0.0493 (6)	
N4	0.8437 (4)	0.1978 (4)	0.1048 (2)	0.0662 (8)	
N5	1.0969 (4)	0.5041 (4)	0.2956 (3)	0.0793 (10)	
C1	0.6221 (4)	0.5515 (4)	0.1613 (3)	0.0531 (8)	
C2	0.5377 (5)	0.6554 (4)	0.1166 (3)	0.0622 (9)	
H2	0.4323	0.6652	0.1285	0.075*	
C3	0.6133 (5)	0.7444 (4)	0.0539 (3)	0.0662 (10)	
H3	0.5592	0.8155	0.0223	0.079*	
C4	0.7674 (5)	0.7276 (4)	0.0385 (3)	0.0690 (10)	
H4	0.8204	0.7867	−0.0038	0.083*	
C5	0.8445 (5)	0.6212 (4)	0.0866 (3)	0.0648 (9)	
H5	0.9503	0.6103	0.0759	0.078*	
C6	0.5531 (4)	0.4478 (4)	0.2283 (3)	0.0603 (9)	
H6	0.4434	0.4439	0.2382	0.072*	
C7	0.5856 (5)	0.2577 (6)	0.3393 (3)	0.0776 (12)	
H7A	0.4603	0.2194	0.3188	0.093*	
H7B	0.6214	0.3151	0.4117	0.093*	
C8	0.6656 (5)	0.1177 (5)	0.3275 (3)	0.0730 (11)	
H8A	0.6415	0.0524	0.3782	0.088*	
H8B	0.6134	0.0506	0.2581	0.088*	
C9	0.9411 (5)	0.2391 (4)	0.4547 (3)	0.0612 (9)	
H9A	0.8978	0.1647	0.4962	0.073*	
H9B	0.9127	0.3402	0.4752	0.073*	
C10	1.1332 (5)	0.2682 (5)	0.4768 (3)	0.0723 (11)	
H10A	1.1776	0.3503	0.4404	0.087*	
H10B	1.1840	0.3088	0.5513	0.087*	
C11	1.1103 (5)	0.0677 (5)	0.3379 (3)	0.0757 (11)	
H11A	1.1471	−0.0283	0.3162	0.091*	
H11B	1.1522	0.1484	0.2998	0.091*	
C12	0.9161 (5)	0.0269 (4)	0.3108 (3)	0.0639 (9)	
H12A	0.8704	−0.0134	0.2360	0.077*	
H12B	0.8735	−0.0576	0.3460	0.077*	
C13	0.8047 (4)	0.1408 (4)	0.0181 (3)	0.0504 (7)	
C14	1.2216 (5)	0.6003 (4)	0.3288 (3)	0.0560 (8)	

### Atomic displacement parameters (Å^2^)

**Table:**

	*U*^11^	*U*^22^	*U*^33^	*U*^12^	*U*^13^	*U*^23^
Zn1	0.0390 (2)	0.0533 (2)	0.0485 (2)	0.00680 (15)	0.01199 (15)	0.00305 (16)
S1	0.0891 (7)	0.0910 (7)	0.0462 (5)	0.0300 (6)	0.0125 (5)	0.0066 (5)
S2	0.0656 (6)	0.0863 (7)	0.0827 (7)	−0.0173 (5)	0.0278 (6)	−0.0084 (6)
O1	0.0664 (17)	0.090 (2)	0.0778 (19)	0.0235 (15)	0.0049 (14)	0.0222 (16)
N1	0.0487 (15)	0.0542 (16)	0.0604 (17)	0.0143 (12)	0.0129 (13)	0.0061 (13)
N2	0.0465 (15)	0.0640 (16)	0.0577 (17)	0.0151 (13)	0.0190 (13)	0.0100 (14)
N3	0.0469 (14)	0.0472 (14)	0.0473 (14)	0.0036 (11)	0.0098 (11)	0.0060 (11)
N4	0.083 (2)	0.0677 (19)	0.0506 (17)	0.0277 (16)	0.0191 (15)	0.0057 (15)
N5	0.0497 (17)	0.077 (2)	0.090 (3)	−0.0094 (16)	−0.0065 (17)	0.0269 (19)
C1	0.0496 (18)	0.0513 (18)	0.0517 (19)	0.0153 (14)	0.0064 (14)	−0.0036 (14)
C2	0.056 (2)	0.059 (2)	0.067 (2)	0.0235 (16)	0.0071 (17)	−0.0023 (17)
C3	0.072 (2)	0.0517 (19)	0.068 (2)	0.0210 (17)	0.0016 (19)	0.0079 (17)
C4	0.070 (2)	0.058 (2)	0.077 (3)	0.0139 (18)	0.017 (2)	0.0159 (19)
C5	0.055 (2)	0.063 (2)	0.080 (3)	0.0176 (17)	0.0196 (18)	0.0153 (19)
C6	0.0466 (18)	0.070 (2)	0.063 (2)	0.0184 (16)	0.0179 (16)	0.0011 (18)
C7	0.053 (2)	0.107 (3)	0.086 (3)	0.018 (2)	0.034 (2)	0.038 (2)
C8	0.051 (2)	0.078 (3)	0.084 (3)	−0.0047 (18)	0.0138 (19)	0.031 (2)
C9	0.074 (2)	0.0553 (19)	0.0471 (19)	0.0103 (17)	0.0135 (17)	0.0017 (15)
C10	0.071 (2)	0.070 (2)	0.057 (2)	0.0043 (19)	−0.0076 (18)	0.0106 (18)
C11	0.075 (3)	0.085 (3)	0.081 (3)	0.035 (2)	0.031 (2)	0.022 (2)
C12	0.082 (3)	0.0477 (18)	0.055 (2)	0.0115 (17)	0.0100 (18)	0.0070 (16)
C13	0.0516 (18)	0.0499 (17)	0.057 (2)	0.0198 (14)	0.0182 (15)	0.0183 (16)
C14	0.061 (2)	0.064 (2)	0.0527 (19)	0.0207 (17)	0.0245 (17)	0.0200 (16)

### Geometric parameters (Å, °)

**Table:**

Zn1—N5	1.951 (3)	C3—C4	1.356 (6)
Zn1—N4	1.959 (3)	C3—H3	0.9300
Zn1—N2	2.051 (3)	C4—C5	1.381 (5)
Zn1—N1	2.273 (3)	C4—H4	0.9300
Zn1—N3	2.279 (3)	C5—H5	0.9300
S1—C13	1.611 (4)	C6—H6	0.9300
S2—C14	1.618 (4)	C7—C8	1.501 (6)
O1—C11	1.401 (5)	C7—H7A	0.9700
O1—C10	1.409 (5)	C7—H7B	0.9700
N1—C5	1.319 (5)	C8—H8A	0.9700
N1—C1	1.339 (4)	C8—H8B	0.9700
N2—C6	1.253 (4)	C9—C10	1.492 (5)
N2—C7	1.462 (5)	C9—H9A	0.9700
N3—C8	1.475 (4)	C9—H9B	0.9700
N3—C9	1.479 (4)	C10—H10A	0.9700
N3—C12	1.486 (4)	C10—H10B	0.9700
N4—C13	1.137 (4)	C11—C12	1.500 (6)
N5—C14	1.122 (4)	C11—H11A	0.9700
C1—C2	1.375 (5)	C11—H11B	0.9700
C1—C6	1.471 (5)	C12—H12A	0.9700
C2—C3	1.374 (6)	C12—H12B	0.9700
C2—H2	0.9300		
			
N5—Zn1—N4	117.35 (16)	N2—C6—H6	120.4
N5—Zn1—N2	126.27 (15)	C1—C6—H6	120.4
N4—Zn1—N2	114.94 (12)	N2—C7—C8	107.8 (3)
N5—Zn1—N1	91.02 (12)	N2—C7—H7A	110.1
N4—Zn1—N1	93.10 (12)	C8—C7—H7A	110.1
N2—Zn1—N1	74.60 (11)	N2—C7—H7B	110.1
N5—Zn1—N3	104.43 (12)	C8—C7—H7B	110.1
N4—Zn1—N3	97.98 (11)	H7A—C7—H7B	108.5
N2—Zn1—N3	79.39 (11)	N3—C8—C7	112.0 (3)
N1—Zn1—N3	153.98 (10)	N3—C8—H8A	109.2
C11—O1—C10	109.3 (3)	C7—C8—H8A	109.2
C5—N1—C1	117.5 (3)	N3—C8—H8B	109.2
C5—N1—Zn1	130.3 (2)	C7—C8—H8B	109.2
C1—N1—Zn1	112.2 (2)	H8A—C8—H8B	107.9
C6—N2—C7	123.2 (3)	N3—C9—C10	112.0 (3)
C6—N2—Zn1	119.9 (2)	N3—C9—H9A	109.2
C7—N2—Zn1	116.5 (2)	C10—C9—H9A	109.2
C8—N3—C9	110.1 (3)	N3—C9—H9B	109.2
C8—N3—C12	107.7 (3)	C10—C9—H9B	109.2
C9—N3—C12	107.8 (3)	H9A—C9—H9B	107.9
C8—N3—Zn1	100.9 (2)	O1—C10—C9	112.2 (3)
C9—N3—Zn1	115.6 (2)	O1—C10—H10A	109.2
C12—N3—Zn1	114.3 (2)	C9—C10—H10A	109.2
C13—N4—Zn1	159.8 (3)	O1—C10—H10B	109.2
C14—N5—Zn1	175.1 (3)	C9—C10—H10B	109.2
N1—C1—C2	123.0 (3)	H10A—C10—H10B	107.9
N1—C1—C6	113.7 (3)	O1—C11—C12	111.9 (3)
C2—C1—C6	123.3 (3)	O1—C11—H11A	109.2
C3—C2—C1	118.2 (3)	C12—C11—H11A	109.2
C3—C2—H2	120.9	O1—C11—H11B	109.2
C1—C2—H2	120.9	C12—C11—H11B	109.2
C4—C3—C2	119.4 (3)	H11A—C11—H11B	107.9
C4—C3—H3	120.3	N3—C12—C11	110.8 (3)
C2—C3—H3	120.3	N3—C12—H12A	109.5
C3—C4—C5	118.9 (4)	C11—C12—H12A	109.5
C3—C4—H4	120.6	N3—C12—H12B	109.5
C5—C4—H4	120.6	C11—C12—H12B	109.5
N1—C5—C4	123.0 (4)	H12A—C12—H12B	108.1
N1—C5—H5	118.5	N4—C13—S1	179.4 (3)
C4—C5—H5	118.5	N5—C14—S2	177.4 (3)
N2—C6—C1	119.3 (3)		
